# Glucocorticoids and 11β-HSD1 are major regulators of intramyocellular protein metabolism

**DOI:** 10.1530/JOE-16-0011

**Published:** 2016-06-01

**Authors:** Stuart A Morgan, Zaki K Hassan-Smith, Craig L Doig, Mark Sherlock, Paul M Stewart, Gareth G Lavery

**Affiliations:** 1 Institute of Metabolism and Systems ResearchInstitute of Biomedical Research, University of Birmingham, Birmingham, UK; 2 Centre for Endocrinology Diabetes and MetabolismBirmingham Health Partners, University of Birmingham, Birmingham, UK; 3 School of MedicineWorsley Building, University of Leeds, Leeds, UK

**Keywords:** glucocorticoid, 11β-HSD1, protein metabolism, Cushing’s syndrome

## Abstract

The adverse metabolic effects of prescribed and endogenous glucocorticoid excess, ‘Cushing’s syndrome’, create a significant health burden. While skeletal muscle atrophy and resultant myopathy is a clinical feature, the molecular mechanisms underpinning these changes are not fully defined. We have characterized the impact of glucocorticoids upon key metabolic pathways and processes regulating muscle size and mass including: protein synthesis, protein degradation, and myoblast proliferation in both murine C2C12 and human primary myotube cultures. Furthermore, we have investigated the role of pre-receptor modulation of glucocorticoid availability by 11β-hydroxysteroid dehydrogenase type 1 (11β-HSD1) in these processes. Corticosterone (CORT) decreased myotube area, decreased protein synthesis, and increased protein degradation in murine myotubes. This was supported by decreased mRNA expression of insulin-like growth factor (IGF1), decreased activating phosphorylation of mammalian target of rapamycin (mTOR), decreased phosphorylation of 4E binding protein 1 (4E-BP1), and increased mRNA expression of key atrophy markers including: atrogin-1, forkhead box O3a (FOXO3a), myostatin (*MSTN*), and muscle-ring finger protein-1 (MuRF1). These findings were endorsed in human primary myotubes, where cortisol also decreased protein synthesis and increased protein degradation. The effects of 11-dehydrocorticosterone (11DHC) (in murine myotubes) and cortisone (in human myotubes) on protein metabolism were indistinguishable from that of CORT/cortisol treatments. Selective 11β-HSD1 inhibition blocked the decrease in protein synthesis, increase in protein degradation, and reduction in myotube area induced by 11DHC/cortisone. Furthermore, CORT/cortisol, but not 11DHC/cortisone, decreased murine and human myoblast proliferative capacity. Glucocorticoids are potent regulators of skeletal muscle protein homeostasis and myoblast proliferation. Our data underscores the potential use of selective 11β-HSD1 inhibitors to ameliorate muscle-wasting effects associated with glucocorticoid excess.

## Introduction

The pathophysiological effects of glucocorticoids (GCs) are well described and impact upon almost all organ systems within the body. This is highlighted in patients with GC excess, Cushing’s syndrome, characterized by central obesity, hypertension, skeletal myopathy, and insulin resistance. In addition, up to 2.5% of the population is taking prescribed GCs, and their side effects represent a considerable clinical burden (Wei *et al.* 2004, ­Hassan-Smith *et al*. 2012).

GC availability and action depend not only on circulating levels, but also on the tissue-specific intracellular metabolism by 11β-hydroxysteroid dehydrogenases (11β-HSDs) (Stewart & Krozowski 1999). Skeletal muscle expresses 11β-hydroxysteroid dehydrogenase type 1 (11β-HSD1), which converts inactive 11-dehydrocorticosterone (11DHC) to active corticosterone (CORT) (cortisone and cortisol in humans, respectively) (Morgan *et al.* 2009). In contrast to 11β-HSD2, which plays no physiological role in this tissue, 11β-HSD1 has been shown to regulate both insulin sensitivity and lipid metabolism of skeletal muscle (Morgan *et al*. 2009, 2013). Furthermore, overexpression has been described in rodent and human models of type 2 diabetes and age-associated muscle weakness (Whorwood *et al.* 2002, Abdallah *et al*. 2005, Zhang *et al.* 2009, Kilgour *et al*. 2013, Hassan-Smith *et al*. 2015), and selective 11β-HSD1 inhibitors have consistently demonstrated an ability to cause insulin sensitization in preclinical and clinical studies (Alberts *et al*. 2003, Bhat *et al*. 2008). We have recently shown that deletion of 11β-HSD1 in mice protects against the adverse side effects associated with both active and inactive circulatory GC excess, including skeletal myopathy; highlighting 11β-HSD1 as a potential therapeutic target in the treatment of Cushing’s syndrome (Morgan *et al.* 2014).

Muscle mass is tightly controlled through the regulation of protein metabolism, myoblast proliferation, and myocyte differentiation – all vital processes in the repair and maintenance of healthy muscle tissue. Mammalian target of rapamycin (mTOR) is a central regulator of protein synthesis, regulating numerous components including the initiation and elongation factors (Thoreen *et al*. 2012). The converse, protein degradation is regulated by components of the E3 ubiquitin proteosomal system (UPS), including muscle-ring finger protein-1 (MuRF1) and atrogin-1, both target cellular proteins to the proteasome for hydrolysis (Lecker *et al*. 2006).

It is well established that GCs drive muscle atrophy through modulation of protein metabolism (McGrath & Goldspink 1982, Savary *et al*. 1998, Biedasek *et al*. 2011). However, the precise molecular mechanisms underpinning GC action, and the role of pre-receptor GC metabolism by 11β-HSD1 in the regulation of intramyocellular protein metabolism and myoblast proliferation have not been fully explored. Therefore, we have characterized the effects of both active and inactive GC treatment, as well as a selective 11β-HSD1 inhibitor, on muscle myotube area, intramyocellular protein synthesis, protein degradation, and myoblast proliferation in a rodent skeletal muscle cell line and human primary muscle cultures.

## Research design and methods

### Cell culture

Murine C2C12 myoblasts (European Collection of Cell Cultures, Salisbury, UK) were grown in DMEM (PAA Laboratories, Somerset, UK) supplemented with 10% fetal bovine serum (FBS) (37°C, 5% CO_2_). Cells were grown to 60–70% confluence before differentiation (initiated by replacing growth media with DMEM with 5% horse serum). After 8 days, myoblasts fuse to form multinucleated myotubes.

Primary human myoblasts were obtained from Promo­Cell (Heidelberg, Germany). Myoblasts were cultured to 80% confluence, as per the manufacturer’s guidelines using the supplied media. Once confluent, growth media was changed to a chemically defined differentiation media (PromoCell) and cells differentiated for 8 days to form myotubes.

Before treatment, all cells were cultured for 4h in serum-free medium without additives. Specific treatments (concentrations and duration) are described in the Results section. The selective 11β-HSD1 inhibitor, PF-877423, was provided through material transfer agreements with Pfizer Global R&D (La Jolla, CA, USA) and its detailed potency has been described previously (Bujalska *et al*. 2008). Sigma supplied all reagents unless otherwise stated. The number of replicates used in each experiment is shown in the figure legends.

### Measurement of myotube area

Myotube cultures were photographed under a phase contrast microscope at 4× magnification following 24h treatments. The areas were measured in a total of 40 myotubes from multiple random fields using ImageJ software (NIH). The observer was blinded to the treatment information at the time of analysis.

### Measurement of protein degradation

Rates of protein degradation were determined by measuring the release of trichloroacetic acid (TCA) and soluble radioactivity from cellular proteins pre-labeled with [^3^H]tyrosine, as described previously (Menconi *et al.* 2008). After completing differentiation, myotubes were labeled with 1.0μCi/mL of l-[3,5-^3^H]tyrosine (PerkinElmer) for 48h in DMEM containing 2% FBS. Cells were then treated for 24h with treatments in DMEM containing 2mM unlabeled tyrosine. The culture medium was then transferred into a microcentrifuge tube containing 100μL of bovine serum albumin (10mg/mL), and TCA was added to a final concentration of 10% (wt/vol). Samples were incubated at 4°C for 1h, followed by centrifugation for 5 min. The supernatant was used for determination of TCA-soluble radioactivity. The protein precipitates were dissolved with a tissue solubilizer (Solvable, PerkinElmer). Cell monolayers were washed with ice-cold phosphate-buffered saline (PBS) and solubilized with 0.5M NaOH containing 0.1% Triton X-100. Radioactivity in the cell monolayer and TCA-soluble and -insoluble fractions were measured using a Packard TRI-CARB 1600 TR liquid scintillation analyzer (Perkin-Elmer). Protein degradation was expressed as the percentage protein degraded over the 24h period and was calculated as 100 times the TCA-soluble radioactivity in the medium divided by the TCA-soluble plus the TCA-insoluble radioactivity in the medium plus the radioactivity in the cell layer.

### Measurement of protein synthesis

Rates of protein synthesis were determined by measuring the [^3^H]tyrosine incorporated into cellular proteins, as described previously (Menconi *et al*. 2008). Differentiated myotubes were treated for 48 h with treatments. During the last hour of incubation, myotubes were incubated with 2.0μCi/mL of l-[3,5-^3^H]tyrosine in DMEM containing 2% FBS and 200mM unlabeled tyrosine. After incubation, the medium was removed and the cell monolayers were harvested in 10% TCA. The TCA-precipitated proteins were washed several times and then solubilized with 0.1N NaOH containing 1% Triton X-100. Radioactivity and protein levels were measured in aliquots from the solubilized proteins.

### Proliferation assay

Myoblast proliferation was assessed using the Celltiter 96 aqueous one solution cell proliferation assay kit (Promega), which is a non-radioactive colorimetric method for determining the number of viable cells in culture. The assay is composed of a tetrazolium compound (3-(4,5-dimethylthiazol-2-yl)-5-(3-carboxymethoxyphenyl)-2-(4-sulfophenyl)-H-tetrazolium, inner salt, MTS) and an electron-coupling reagent (phenazine methosulfate (PMS)). Myoblasts were trypsinized and counted, before seeding at a concentration of approximately 1000 cells per well in 96-well plates (final volume: 100μL), and incubated at 37°C, 5% CO_2_ for 24h. Treatments were added to the culture media and the cells were further incubated at 37°C, 5% CO_2_ for 48h. The proliferation assay was subsequently performed according to the manufacturer’s protocol. Incubation with IGF1, a known activator of myoblast proliferation (Milasincic *et al*. 1996), was used as a positive control in these experiments. Selected results generated using the above methodology were validated using a 5-bromo-2′-deoxyuridine (BrdU) Cell Proliferation Assay Kit (Cell Signaling). The assay was performed according to the manufacturer’s protocol using similar seeding densities and conditions described above.

### 11β-HSD1 enzyme assays

Briefly, intact cell monolayers were incubated for 2h at 37°C with 100nM 11DHC and tracer amounts of [^3^H]-11DHC (synthesized in-house (Bujalska *et al*. 2002)). Steroids were then extracted from the culture media using dichloromethane, separated using a mobile phase consisting of ethanol and chloroform (8:92) by thin layer chromatography and scanned using a Bioscan 3000 image analyzer (Lablogic, Sheffield, UK). Protein levels were assayed using a commercially available kit (Bio-Rad), and activity expressed as pmol of CORT generated/mg of protein/h.

### RNA extraction and real-time PCR

Total RNA was extracted from tissue and cells using the Tri-Reagent system. RNA integrity was assessed by electrophoresis on 1% agarose gel. Concentration was determined spectrophotometrically at an optical density of (OD)_260_. In a 50μL volume, 500ng of total RNA were incubated with 250μM random hexamers, 500μM dNTPs, 20U RNase inhibitor, 63U multiscribe reverse transcriptase, 5.5mM MgCl and 1× reaction buffer. The reverse transcription reaction was carried out at 25°C for 10min, 48°C for 30min before the reaction was terminated by heating to 95°C for 5min. mRNA levels were determined using an ABI 7500 sequence detection system (Applied Biosystems). Reactions were performed in singleplex in 10μL volumes on 96-well plates in a reaction buffer containing 2× TaqMan Universal PCR Master Mix (Applied Biosystems). Primers and probes were supplied by Applied Biosystems as premade ‘assay on demands’. All reactions were normalized against the housekeeping gene 18S rRNA, provided as a pre-optimized control probe. All target genes were labeled with 6-carboxyfluorescein (6-FAM), and the reference gene with 2′-chloro-7′phenyl-1,4-dichloro-6-carboxy-fluorescein (VIC). The reaction conditions were as follows: 95°C for 10min, then 40 cycles of 95°C for 15s, and 60°C for 1min. Data were obtained as Ct values (Ct=cycle number at which logarithmic PCR plots cross a calculated threshold line) and used to determine ΔCt values (ΔCt=(Ct of the target gene) – (Ct of the reference gene)). Data were expressed as arbitrary units using the following transformation (arbitrary units (AU)=1000 × (2^−Δct^)).

### Protein extraction and immunoblotting

Monolayers of cells were placed on ice, washed with cold PBS, and then scraped into 100μL of radioimmunoprecipitation assay (RIPA) buffer. For both tissue and cultured cell homogenates, samples were incubated at −80°C (10min) on ice (30min), and centrifuged at 4°C (10min, 10,000g). The supernatant was transferred to a fresh tube and total protein concentration was determined by a commercially available assay (Bio-Rad Laboratories). An amount of 30–40μg of protein was resolved on to an SDS-PAGE (acryl-amide percentage varied according to protein size). Proteins were transferred onto nitrocellulose membrane, Hybond ECL (GE Healthcare). Primary (anti-mTOR,anti-pSer2448 mTOR, anti-4E binding protein 1 (anti-4E-BP1), anti-pThr37/46 4E-BP1, and anti-MuRF1 were purchased from Cell Signaling) and secondary antibodies (Dako) were used at a dilution of 1/2000. Membranes were re-probed for β-actin or α-tubulin and primary and secondary antibodies used at a dilution of 1/5000 (Abcam). Bands were visualized using ECL detection kit (GE Healthcare) and quantified using ImageJ software.

### Statistical analysis

Statistical comparisons were performed using SigmaStat 3.1 (Systat Software, Inc., Point Richmond, CA, USA). Data are presented as mean±s.e.m. with statistical significance defined as *P<*0.05. One- or two-way ANOVA followed by Bonferroni’s multiple comparison *post hoc* test was used to compare treatments. Statistical analysis on real-time PCR data was performed on ΔCt values and not fold-changes or AU.

## Results

**Figure 1:**
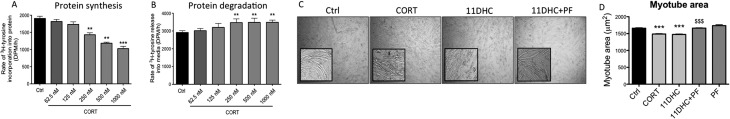
Treatment of C2C12 myotubes with corticosterone (CORT: 62.5–1000nM, 24h) decreased [^3^H]tyrosine incorporation into cellular proteins (protein synthesis). (A) Paralleled by increased TCA-soluble radioactivity released into media from cells pre-labeled with [^3^H]tyrosine (protein degradation). (B) Data analyzed using one-way ANOVA. C2C12 myotube area was decreased following treatment with CORT (250nM, 24h) and 11-dehydrocorticosterone (11DHC, 250nM, 24h). (C) The selective 11β-HSD1 inhibitor, PF-877423 (PF: 2.5μM, 24h), blocked the effects of 11DHC. Myotube area quantified from images using ImageJ software and analyzed using two-way ANOVA (C). Data expressed as mean±s.e.m. of *n*=6 experiments. (**P<0.01, ***P<0.001 vs ctrl, ^$$$^*P*<0.001 vs 11DHC).

Rates of protein synthesis and protein degradation were assessed in C2C12 myotubes by measuring [^3^H]tyrosine incorporation into cellular proteins and measuring TCA-soluble radioactivity released into the culture media from cellular proteins pre-labeling with [^3^H]tyrosine, respectively. CORT (62.5–1000nM, 24h) dose dependently decreased protein synthesis (Fig. 1A), while concomitantly increased protein degradation (Fig. 1B).

Consistent with the functional data, both CORT and 11DHC treatment (250nM, 24h) decreased C2C12 myotube area (Fig. 1C and D). Importantly, the effects of 11DHC were blocked by co-incubation with the selective 11β-HSD1 inhibitor, PF-877423 (2.5μM, 24h) (Fig. 1C and D). A concentration of 250nM was used, as this was the lowest concentration of CORT that resulted in statistically significant changes in rates of both protein synthesis and protein degradation.

**Figure 2:**
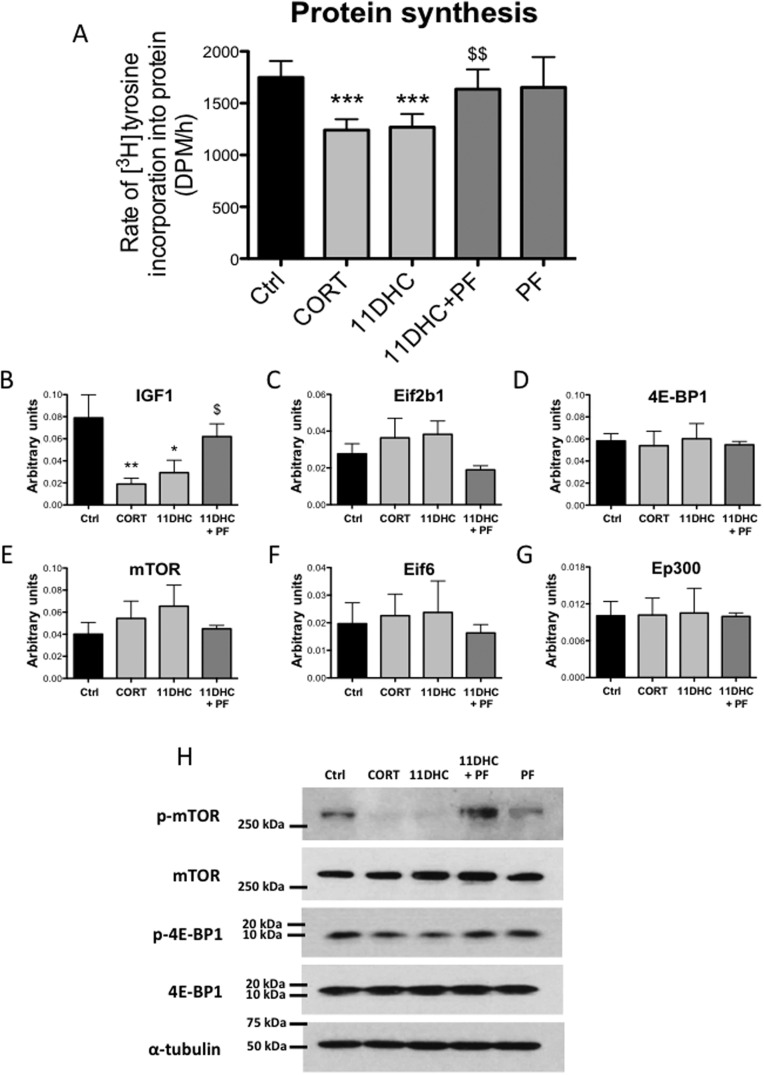
[^3^H]tyrosine incorporation into cellular proteins in C2C12 myotubes (protein synthesis) was decreased by both corticosterone (CORT: 250nM, 24h) and 11-dehydrocorticosterone (11DHC: 250nM, 24h). The selective 11β-HSD1 inhibitor, PF-877423 (PF: 2.5μM, 24h), blocked the effects of 11DHC (A). Both CORT (250nM, 24h) and 11DHC (250nM, 24h) decreased IGF1 mRNA expression in C2C12 myotubes (B), but Eif2b1 (C), 4E-BP1 (D), mTOR (E), Eif6 (F), and Ep300 (G) mRNA expression was unchanged. Serine-2448 phosphorylation of mTOR and threonine-37/46 phosphorylation of 4E-BP1 were decreased by both CORT (250nM, 24h) and 11DHC (250nM, 24h) in C2C12 myotubes, without effecting total protein levels. The effects of 11DHC on IGF1 expression, p-mTOR and p-4E-BP1 were reversed by PF-877423 (PF: 2.5μM, 24h) (B and H). Data expressed as mean ±s.e.m. of *n*=6 experiments, and analyzed using two-way ANOVAs. Western blot analyses were carried out on at least three different preps. (*P<0.05, **P<0.01, ***P<0.001 vs ctrl, ^$^*P*<0.05, ^$$^*P*<0.01 vs 11DHC).

Both CORT and 11DHC treatment (250nM, 24h) decreased [^3^H]tyrosine incorporation into cellular ­proteins (functional assessment of protein synthesis) in C2C12 myotubes (Fig. 2A), with the effects of 11DHC reversed by PF-877423 (2.5μM, 24h). Gene expression analysis revealed decreased mRNA expression of ­insulin-like growth factor (IGF1) (a positive regulator of muscle size and mass) following both CORT and 11DHC treatment (Fig. 2B). By contrast, the mRNA expression of the following key genes regulating protein metabolism were unchanged: eukaryotic translation initiation factor 2B (Eif2B1) (a GDP exchange factor important for initiation of protein synthesis), eukaryotic translation initiation factor 4E-BP1 (a repressor of protein translation), mTOR (a serine/threonine kinase that senses nutritional/environmental cues and couples that signals to regulate protein metabolism), eukaryotic translation initiation factor 6 (EiF6) (forms a complex with 40s ribosomal subunit involved in translation initiation), and **histone acetyltransferase p300 (**EP300) (a key activator of translation) (Fig. 2C, D, E, F and G). Consistent with decreased protein synthesis, a trend toward decreased activating serine-2448 phosphorylation of mTOR and threonine-37/46 phosphorylation of 4E-BP1 was observed following CORT and 11DHC treatment, without effecting total protein levels (Fig. 2H). Importantly, PF-877423 reversed the effect of 11DHC on IGF1 mRNA expression, mTOR serine-2448 phosphorylation and 4E-BP1 threonine-37/46 phosphorylation (Fig. 2A, B and H). PF-877423 treatment alone (2.5μM, 24h) was without effect on all parameters assessed.

**Figure 3:**
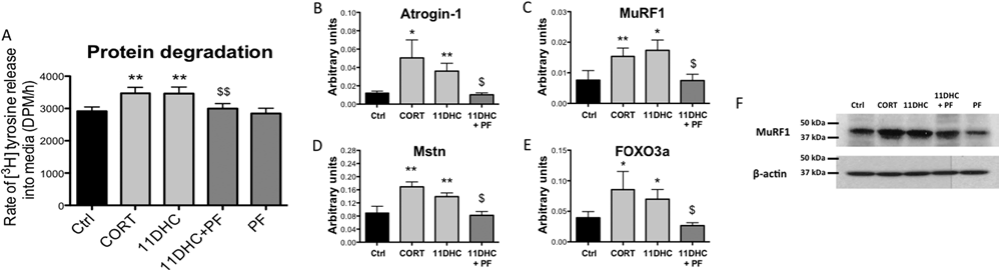
TCA-soluble radioactivity released into the culture media from C2C12 myotubes pre-labeled with [^3^H]tyrosine was increased by both corticosterone (CORT: 250nM, 24h) and 11-dehydrocorticosterone (11DHC: 250nM, 24h). The selective 11β-HSD1 inhibitor, PF-877423 (PF: 2.5μM, 24h), blocked the effects of 11DHC (A). Both CORT (250nM, 24h) and 11DHC (250nM, 24h) increased atrogin-1 (B), MuRF1 (C), Mstn (D), and FOXO3a (E) mRNA expression in C2C12 myotubes. Total protein levels of MuRF1 were also increased by both CORT and 11DHC (F). The effects of 11DHC on atrogin-1, MuRF1, Mstn, and FOXO3a mRNA expression as well as MuRF1 total protein levels was reversed by PF-877423 (PF: 2.5μM, 24h) (B, C, D, E and F). Data expressed as mean ±s.e.m. of *n*=6 experiments, and analyzed using two-way ANOVAs. Western blot analyses were carried out on at least three different preps. (*P<0.05, **P<0.01 vs ctrl, ^$$^*P*<0.01 vs 11DHC).

Both CORT and 11DHC treatment (250nM, 24h) increased the TCA-soluble radioactivity released into the culture media from C2C12 myotube cellular proteins pre-labeled with [^3^H]tyrosine (functional assessment of protein degradation), with the effects of 11DHC reversed by PF-877423 (2.5μM, 24h) (Fig. 3A). In agreement, the mRNA expression of key muscle atrophy markers including: atrogin-1, MuRF1 (both E3 ubiquitin ligases), myostatin (*MSTN*) (a negative regulator of muscle mass), and forkhead box O3a (FOXO3a) (a transcription factor that regulates muscle mass), as well as MuRF1 total protein levels, were increased by both CORT and 11DHC, with PF-877423 blocking the effects of 11DHC on all these readouts (Fig. 3B, C, D, E and F). PF-877423 treatment alone (2.5 , 24h) was without effect on all parameters assessed.

**Figure 4:**
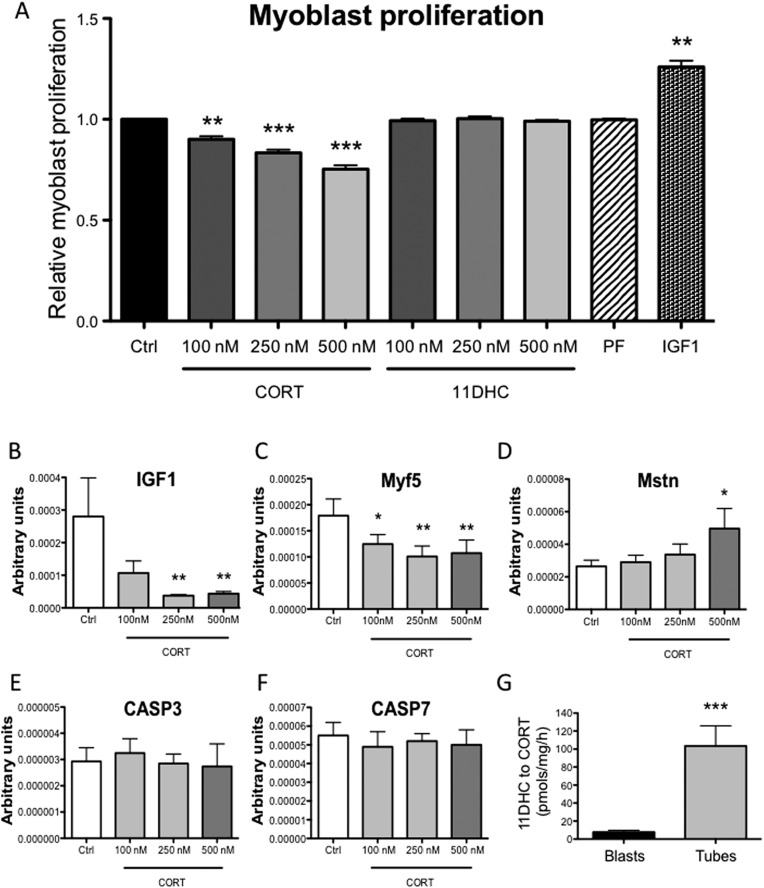
C2C12 myoblast proliferation was decreased by corticosterone (CORT: 100–500nM, 48h), but not 11-dehydrocorticosterone (11DHC: 100–500nM, 48h), in a concentration-dependent manner (A). C2C12 myoblasts treated with CORT (100–500nM, 24h) had deceased mRNA expression of IGF1 (B) and Myf5 (C), while Mstn expression was increased (D), whereas CORT was without effect on CASP3 (E) and CASP7 (F) expression. Low oxoreductase activity of 11β-HSD1 (conversion of inactive 11DHC to active CORT) was detected in C2C12 myoblasts, with activity increasing across myocyte differentiation (G). Data expressed as mean ±s.e.m. of *n*=6 experiments, and analyzed using one-way ANOVAs. (*P<0.05, **P 0.01, ***P 0.001 vs ctrl).

Myoblast proliferation is vital for the repair and maintenance of healthy muscle tissue. As such, we assessed the impact of both CORT and 11DHC on C2C12 myoblast proliferation (Fig. 4A). CORT (100–500nM, 48h) decreased myoblast proliferation in a concentration-­dependent manner. This was consistent with CORT-mediated decreased expression of IGF1 and myogenic factor 5 (MYF5) (both of which positively regulate myoblast proliferation) (Fig. 4B and C), whereas MSTN (a negative regulator of myoblast proliferation) expression was increased following CORT treatment (Fig. 4D). Importantly, we observed no difference in CASP3 or CASP7 expression (key apoptotic effectors) (Fig. 4E and F), suggesting that the observed CORT-mediated decrease in myoblast proliferation was not simply down to increased myoblast apoptosis.

In contrast to CORT, 11DHC (100–500nM, 48h) (which relies on activation to CORT by 11β-HSD1) was without effect upon C2C12 myoblast proliferation (Fig. 4A), consistent with the 30-fold lower 11β-HSD1 oxoreductase activity (11DHC to CORT) in myoblasts compared with myotubes (Fig. 4G). Incubation with IGF1 (10ng/mL, 48h), a known activator of myoblast proliferation, was used as a positive control in these experiments (Fig. 4A). The C2C12 myoblast proliferation data presented in Fig. 4 were measured using MTS and validated using BrdU (Supplementary Fig. 1, see section on supplementary data given at the end of this article).

**Figure 5:**
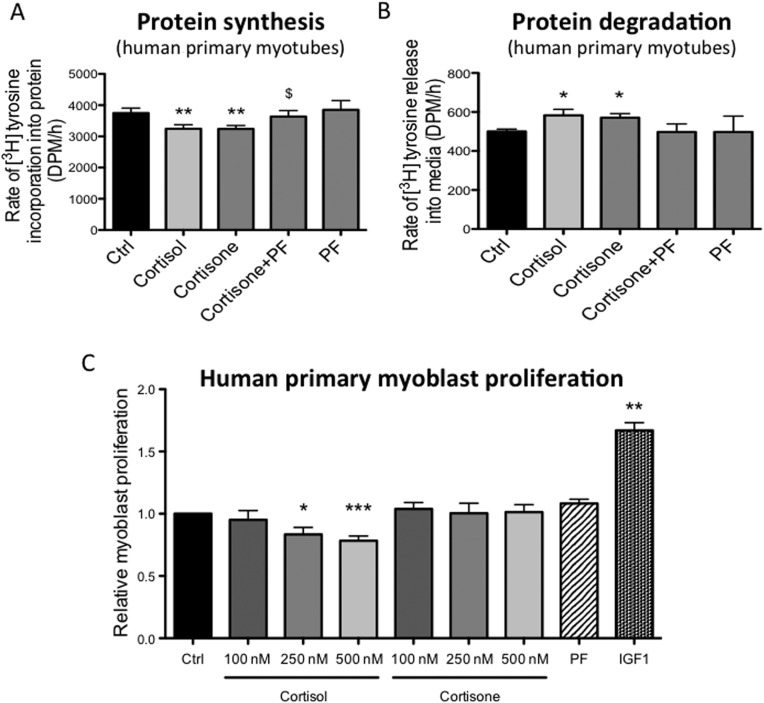
Treatment of differentiated human primary myotubes with cortisone (250nM, 24h) decreased [^3^H]tyrosine incorporation into cellular proteins (protein synthesis) (A), paralleled by increased TCA-soluble radioactivity released into the culture media from cells pre-labeled with [^3^H]tyrosine (protein degradation) (B). The selective 11β-HSD1 inhibitor, PF-877423 (PF: 2.5μM, 24h), blocked the effects of cortisone on both protein synthesis and protein degradation. Proliferation of human primary myoblasts was decreased by cortisol (100–500nM, 48h) in a concentration-dependent manner, but not cortisone (100–500nM, 48h), (C). Data expressed as mean±s.e.m. of *n*=6 experiments, and analyzed using one- or two-way ANOVAs. (*P<0.05, **P<0.01, ***P<0.001 vs ctrl, ^$^*P*<0.05 vs cortisone).

To test whether these observations are relevant in a human setting, we treated human primary skeletal myotubes with cortisone (250nM, 24h). Consistent with our findings in the C2C12s, cortisone decreased protein synthesis ([^3^H]tyrosine incorporation into cellular proteins) (Fig. 5A) and increased protein degradation (radioactivity release into the culture media from cellular proteins pre-labeled with [^3^H]tyrosine) (Fig. 5B). The effects of cortisol (250nM, 24h) on protein synthesis and protein degradation in human primary myotubes were identical to that of cortisone (data not shown). Crucially, the effects of cortisone on protein synthesis and protein degradation were blocked by PF-877423 (2.5μM, 24h) (Fig. 5A and B). In agreement with the C2C12 data, cortisol (and not cortisone) was effective at suppressing myoblast proliferation (Fig. 5C).

## Discussion

GC excess leads to skeletal muscle atrophy, however, the precise molecular mechanisms underpinning this observation is not completely understood. This has implications not only for the 1–2% of the population taking prescribed GCs (Wei *et al*. 2004), but also where elevated endogenous GC levels contribute to the muscle atrophy seen with starvation (Wing & Goldberg 1993), sepsis (Tiao *et al*. 1996), and metabolic acidosis (May *et al*. 1986). Here, we have characterized the impact of GCs and pre-receptor GC metabolism upon intramyocellular protein metabolism and myoblast proliferation.

We have shown that GCs decrease the rate of protein synthesis in both mouse C2C12 and human primary myotubes, in agreement with previously published reports (McGrath & Goldspink 1982, Savary *et al*. 1998, Shah *et al*. 2000). Decreased activating serine-2448 ­phosphorylation of mTOR, a master regulator of protein metabolism, underpinned this finding. However, due to the vast number of environmental and nutritional cues that modulate mTOR activity, the mechanism(s) driving GC-mediated suppression of mTOR are likely to be complex (Laplante & Sabatini 2012). Despite this, we did observe decreased IGF1 mRNA expression in C2C12 myotubes. IGF1 is synthesized and secreted by skeletal muscle and acts autocrine, paracrine, and endocrine to stimulate mTOR, acting via AKT/PI3K pathway (Bodine *et al*. 2001, Rommel *et al*. 2001). Importantly, we have previously shown GCs to decrease insulin/IGF1-stimulated activation of AKT (Morgan *et al*. 2009), consistent with the observed reduction in mTOR serine-2448 phosphorylation and suppressed protein synthesis reported here.

In agreement with attenuated protein synthesis, we observed a trend toward decreased 4E-BP1 threonine-37/46 phosphorylation following GC treatment. 4E-BP1 directly interacts with eukaryotic translation initiation factor 4E (eIF4E), which is a limiting component of the multisubunit complex that recruits 40S ribosomal subunits to the 5′ end of mRNAs to be translated (Pause *et al*. 1994, Youtani *et al*. 2000). Phosphorylation at threonine-37/46 facilitates in its dissociation from eIF4E, allowing activation of mRNA translation. Interestingly, there is evidence that mTOR directly phosphorylates 4E-BP1 at threonine-37/46 (Gingras *et al*. 1999). As such, the observed decrease in mTOR activation following GC treatment could explain the hypophosphorylated state of 4E-BP1, keeping this suppressor of RNA translation firmly associated with eIF4E, thereby attenuating protein synthesis.

Concomitant with suppressed protein synthesis, GCs augmented protein degradation in both mouse C2C12 and human primary myotubes, consistent with previously published reports (McGrath & Goldspink 1982, Biedasek *et al*. 2011). This was underpinned by increased mRNA and protein expression of the key E3 ubiquitin ligases: MuRF1 and atrogin-1. In addition, the mRNA expression of *MSTN*, a known negative regulator of muscle size and mass, was decreased by GC treatment. Importantly, the GC-mediated decrease in protein synthesis and increased protein degraded resulted in decreased C2C12 myotube area, consistent with the known muscle atrophying effects of GCs. Taken together, these data provide new mechanistic insight into the manner in which GCs mediate muscle-specific side effects associated with GC excess.

Previously, Biedasek and coworkers demonstrated that the *non-selective* HSD inhibitor, carbenoxolone, blocked cortisone-induced increase in protein degradation in human and murine myocytes induced by cortisone (Biedasek *et al*. 2011). In the current study, we have shown that the *selective* 11β-HSD1 inhibitor, PF-877423, not only blocks 11DHC/cortisone-mediated increased protein degradation, but also 11DHC/cortisone-mediated suppression of protein synthesis in both C2C12 and human primary myotubes. These findings were validated at a molecular level, where 11β-HSD1 inhibition also blocked the gene expression changes and post-translational protein phosphorylation events mediated by 11DHC in C2C12 myotubes. As the net effect of these functional changes was to reverse 11DHC-mediated C2C12 myotube atrophy, these results underscore the potential for a selective 11β-HSD1 inhibitor for the treatment of muscle atrophy induced by GC excess.

Regulation of muscle mass is not only governed by protein turnover, but also by myoblast proliferation is also a key ­factor in the repair and maintenance of healthy muscle tissue. Although previous studies have identified the highly potent synthetic GC, dexamethasone, to inhibit myoblast proliferation (te Pas *et al*. 2000, Dong *et al*. 2013), the impact of endogenous GCs, and the role of pre-receptor GC metabolism by 11β-HSD1 plays on myoblast proliferation has not been explored. In the present study, we found that active GCs (CORT/cortisol) to inhibit both rodent and human myoblast proliferative capacities, in agreement with the known effects of dexamethasone (te Pas *et al.* 2000, Dong *et al*. 2013). However, we found treatment with the inactive GCs (11DHC/cortisone) to be without effect upon myoblast proliferation, which a finding corroborated by 30-fold lower oxoreductase activity of 11β-HSD1 (11DHC to CORT) in myoblasts compared with myotubes. As such, it is plausible that GCs reactivated by 11β-HSD1 in mature myotubes regulate the proliferative capacity of undifferentiated myoblast pools located proximally in the muscle bed. This may represent an additional mechanism by which GCs and pre-receptor GC metabolism negatively regulate muscle mass.

Classical physiology studies have found transient GCs exposure to improve skeletal muscle performance (Schakman *et al*. 2009). These ergonomic effects are mediated by the induction of the metabolic transcription factor Krüppel-like factor 15 (KLF15) (Morrison-Nozik *et al*. 2015), defining a downstream pathway distinct from that resulting in GC-related muscle atrophy. These findings highlight the complex role GCs play in regulating of muscle physiology. Although, the transient effects of GCs have not been explored as part of the present study, investigation into the role of pre-receptor GC metabolism in the regulation of these ergogenic effects would be of great interest in future studies.

In conclusion, we have demonstrated that GCs are potent regulators of skeletal muscle protein homeostasis and myoblast proliferation. In addition, we have provided novel mechanistic insights into how GCs regulate these important metabolic pathways *in vitro*. Crucially, our data underscore the potential for the use of a selective 11β-HSD1 inhibitor to ameliorate muscle-wasting effects associated with GC excess in exogenous and endogenous Cushing’s syndrome, starvation, sepsis, and metabolic acidosis.

## Supplementary data

This is linked to the online version of the paper at http://dx.doi.org/10.1530/JOE-16-0011.

## Declaration of interest

The authors declare that there is no conflict of interest that could be perceived as prejudicing the impartiality of the research reported.

## Funding

This work has been supported by the Biotechnology and Biomedical Sciences Research Council (ref. BB/G023468/1, (G G L); ref. BBB/S/M/2006/13045 (S A M)), ERC Advanced Research Grant (PRECORT) (P M S), Wellcome Trust Senor Research Fellowship (G G L) and a BMedSci final year project grant provided by the University of Birmingham.

## Author contribution statement

S A M, Z K H S, and C L D generated data for the manuscript. S A M, Z K H S, C L D, M S, P M S, and G G L all contributed to the writing of the manuscript. Study protocols were devised by S A M, Z K H S, and G G L. Project and hypothesis conceived by S A M and Z K H S.
